# Development of a core outcome set for diabetes after pregnancy prevention interventions (COS-DAP): a study protocol

**DOI:** 10.1186/s13063-018-3072-y

**Published:** 2018-12-29

**Authors:** Karoline Kragelund Nielsen, Sharleen O’Reilly, Nancy Wu, Kaberi Dasgupta, Helle Terkildsen Maindal

**Affiliations:** 1 Health Promotion Research, Steno Diabetes Center Copenhagen, Niels Steensens Vej 6, 2820 Gentofte, Denmark; 2 Section of Epidemiology, Department of Public Health, University of Copenhagen, Oestre Farimagsgade 5, 1014 Copenhagen K, Denmark; 3 School of Agriculture and Food Science, University College Dublin, Belfield, Dublin 4, Ireland; 4 Centre for Outcomes Research and Evaluation (CORE), Research Institute of the McGill University Health Centre, 5252 boul de Maisonneuve Ouest, Office 3E.09, Montréal, QC H4A 3S5 Canada; 5 Department of Medicine, McGill University, 5252 boul de Maisonneuve Ouest, Office 3E.09, Montréal, QC H4A 3S5 Canada; 6 Section for Health Promotion and Health Services Research, Department of Public Health, Aarhus University, Bartholins Allé 2, bdg. 1260, 218, 8000 Aarhus C, Denmark

**Keywords:** Gestational diabetes, Diabetes prevention, Core outcome set, Behavior change interventions

## Abstract

**Background:**

Gestational diabetes mellitus (GDM) increases the risk of adverse short- and long-term outcomes, including development of type 2 diabetes. The US Diabetes Prevention Program demonstrates this risk can be halved with an intensive health behavior change intervention in women with pre-diabetes averaging 12 years since a GDM pregnancy. In recent years, the number of studies looking at changing the behaviors of women with previous GDM closer to the time of delivery has steadily grown, but reported outcomes vary and most studies are not long enough or large enough to examine incident diabetes. This initiative aims to develop a core outcome set (COS) for interventions seeking to prevent diabetes after pregnancy (DAP) in both women with prior GDM and their families.

**Methods:**

The COS-DAP project will use established COS methodology, in four stages: (1) a systematic literature review of DAP prevention intervention studies following GDM; (2) discussion and cataloguing of outcomes measured and implementation components at an investigator meeting; (3) a two-round online Delphi survey aimed at prioritizing the identified outcomes; and (4) a consensus meeting with key stakeholders to review, discuss, and refine suitable COS measures, using nominal group technique.

**Discussion:**

COS-DAP aims to develop a COS for health behavior change interventions to prevent DAP. The COS is expected to enhance opportunities for comparison of future studies and allow for better synthesis of the effects. The inclusion of multiple stakeholder perspectives will increase the final COSs applicability and relevance.

**Trial registration:**

Comet Initiative, COMET 1083; PROSPERO, CRD42018084853. Registered in prospero on 03/01/2018.

**Electronic supplementary material:**

The online version of this article (10.1186/s13063-018-3072-y) contains supplementary material, which is available to authorized users.

## Background

Gestational diabetes mellitus (GDM) is one of the most common pregnancy complications affecting around 5–15% of pregnancies [1–3]. Studies show that GDM increases the risk of adverse short- and long-term health outcomes in both the woman and her offspring. A previous meta-analysis reported that women with a history of GDM had a sevenfold increased risk of developing type 2 diabetes [4]. A more recent meta-analysis suggests that the adjusted risk is 18-fold higher [5]. Offspring of women with GDM are also at elevated risk of developing diabetes [6] and we have demonstrated that GDM in mothers predicts incident diabetes in fathers [7]. Thus, developing effective interventions is imperative as these interventions have the potential to have a lasting impact on the health of the whole family.

While few have intervened at the family level, the evidence from the US Diabetes Prevention Program (DPP) trial indicates that intensive health behavior change intervention can reduce the progression to diabetes by 58% in women with a GDM history [8]. These women were however an average of 12 years after their first pregnancy complicated by GDM and had already developed impaired glucose tolerance, indicating they were further along the GDM to diabetes trajectory. In contrast, the majority of diabetes after pregnancy (DAP) occurs during the first five postpartum years [9]. Our recent cohort study demonstrates that among women who develop DAP, the median time to DAP after GDM was approximately five years compared with 10 years in women without GDM or gestational hypertension [10]. These early postpartum years present women with additional challenges through the combined pressures of new motherhood and work-related responsibilities [11–13], which can result in lower prioritization and less perceived time for engagement in DAP prevention activities.

In the years following the US DPP, many investigators have conducted DAP prevention studies focusing on women during pregnancy or within the first five years, with an eye to aligning intervention frequency, intensity, and design to the day-to-day realities of life with a young family [14]. Unfortunately, the reported outcomes from these studies differ widely and the lack of consistency limits the understanding of behavior change mechanisms and intervention effects. The amount of information recorded on the wider context in which the intervention is implemented is limited as is use of theoretical frameworks for behavior change. Consequently, it remains unclear which outcome measures are the most important to include in health behavior change intervention studies, whether these outcomes should be adapted as the woman and her family grow older, and which contextual factors influence intervention effectiveness.

The development of a core outcome set (COS) for health behavior change interventions in women with previous GDM is urgently needed. A COS is a standardized set of outcomes to be reported across all trials within a specific area [15]. The importance of developing COSs is specifically promoted within the CROWN initiative led by the editors of more than 80 journals aiming to harmonize outcome reporting for women’s health, thus enabling more effective evidence synthesis (http://www.crown-initiative.org/journals/) [16]. Our project seeks to develop a COS for intervention studies with the objective of DAP prevention in women with prior GDM and their families.

## Methods

**Fig. 1:**
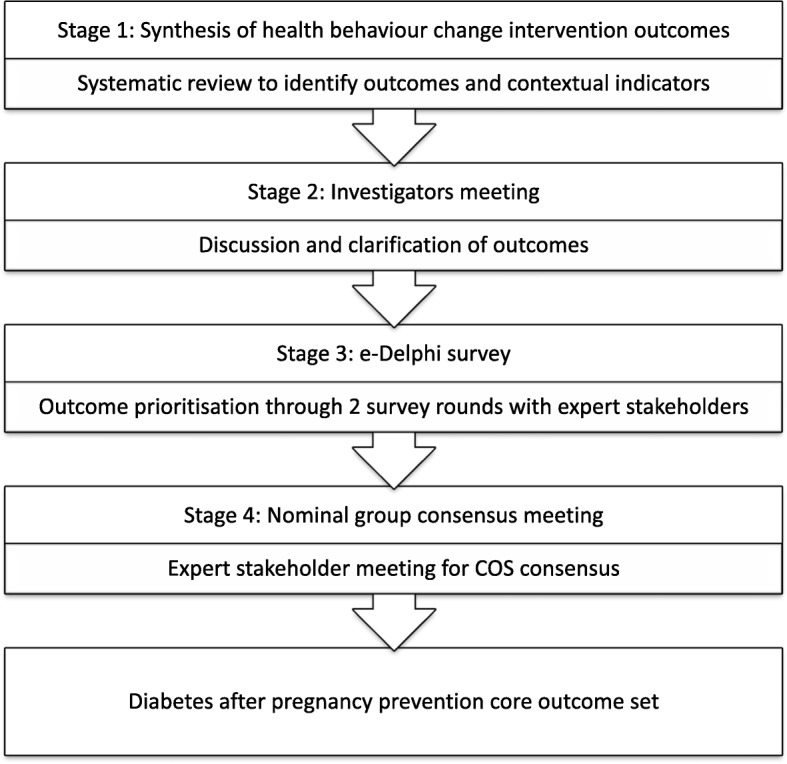
Core set of outcomes development overview for COS-DAP project

The COS-DAP project was launched in October 2017 and adheres to the Core Outcomes Measures in Effective Trials (COMET) initiative and the Core Outcome Set – Standards for Reporting (COS-STAR) statement [17]. The study has been registered in COMET (1083) and has four stages (Fig. 1):

1. A systematic literature review to identify core outcomes in DAP prevention interventions and contextual indicators that may influence both recruitment and impact;
2. A discussion and outcome clarification meeting to inform the Delphi survey;
3. A two-round Delphi survey to prioritize the identified outcomes and contextual indicators; and
4. A nominal group technique consensus meeting with key stakeholders to identify the COS and refine suitable measures to assess them.

### Stage 1: Synthesis of health behavior change intervention outcomes

The identification of the outcomes will be conducted via systematic literature review of the existing literature.

#### Search strategy

The search will use the electronic databases Embase, OVID Medline, CINAHL, Cochrane Central Register of Controlled Trials, and Cochrane Pregnancy and Childbirth Group Trials Register. A combination of a number of terms, including MeSH terms, will be employed, including “gestational diabetes” and/or “diabetes in pregnancy,” “postpartum period,” “diabetes reduction,” and/or “diabetes prevention,” as well as terms related to the intervention (e.g. “diet,” “body mass index,” “weight reduction,” “exercise,” “health behavior change,” “cognitive therapy”). The search will be limited to published literature and English language, but there will be no restrictions in terms of publication date or study intervention type. The systematic review has been registered at PROSPERO (CRD42018084853). The PROSPERO and COMET websites were searched to identify potentially similar research and determine the applicability of the search strategy. A single COMET study was sufficiently similar to contact the lead investigator (reference no. 686); however, following discussion, the investigators felt the focus of the COS was sufficiently different to merit the COS-DAP project continuing.

#### Study selection, eligibility, and data extraction

Eligible studies will report outcomes in the postpartum period in women with a history of GDM and/or their extended family. Outcomes related to the infant or father will also be selected for review. In order to be eligible for inclusion, studies have to be randomized controlled trials (RCT), pre- and post-intervention studies, multi-center studies, clinical trials, comparative studies, evaluation studies, and intervention protocols. Reviews, case reports, meta-analysis, systematic reviews, observational studies, letters, guidelines, commentaries, historical articles, and editorials will be excluded. Interventions designed to change health behaviors such as diabetes screening and lifestyle outcomes will be included, whereas studies focusing on pre-pregnancy GDM outcomes, pharmacological treatments, or supplement trials will be excluded.

Two reviewers will independently screen all articles by title and abstract. Full-text articles will be reviewed in duplicate as a minimum to determine whether suitable for inclusion. Data will be abstracted and recorded on a standardized form. Any disagreement on the eligibility at any stage will be resolved through discussion and consensus with the research team. The Preferred Reporting Items for Systematic Reviews and Meta-Analyses (PRISMA) guideline will be followed in the documentation of the process.

The types of contextual data recorded will be guided by the Penetration, Implementation, Participation, and Effectiveness (PIPE) framework and the Context and Implementation of Complex Interventions (CICI) framework [18, 19]. PIPE is designed to evaluate real-world program and product design elements; as such, it is useful a guide to assess the net impact of health improvement programs [18] and thus to increase the relevance of our findings to policy and practice. There are three dimensions within the CICI framework [19]. Three levels exist for any domain: macro, meso, and micro; these can be used to add relevant detail to a domain. The extent of the usefulness of the CICI and PIPE frameworks data will be evaluated at stage 2 and may or may not be included in subsequent stages pending this evaluation.

#### Data analysis

The characteristics of each included study will be presented in tables and descriptively. Outcomes will be divided into domains based on thematic exploration of the outcomes identified and grouping them thematically to represent overarching categories. For example, we expect several measures of diabetes risk to be outcomes. The stage 2 process will see these outcomes narratively synthesized into a long-list of health behavior change intervention outcomes and their associated measurement tools or contextual factors where relevant. Analyses related to the outcomes, the context, the process, and the behavioral change frameworks are planned. The exact identification of domains and categorization of outcomes and contextual factors will be agreed upon during the discussion meeting in the next stage of the development.

### Stage 2: Investigators meeting

The meeting will enable a roundtable discussion among the members of the research team on the systematic review findings and clarification of identified outcomes and outcome domains. The participants at this meeting (SOR, KD, KKN, HTM) will involve representation from the following stakeholder groups: public health; health promotion; dietetics; and internal medicine. For this meeting, all the outcomes identified in the systematic review will be listed and the grouping into domains will be discussed and executed. Outcomes related to the mother, father, and child will be discussed separately in addition to considering biological, behavioral, health outcomes, and contextual measures. Further, the strength of the evidence and the knowledge gaps will be reviewed and discussed.

The meeting will also be used to plan the e-Delphi surveys, including the development and refinement of question stems relevant to each section. Once these have been drafted, they will be consolidated into final draft surveys.

### Stage 3: e-Delphi survey

In order to prioritize the outcomes and contextual factors identified during stages 1 and 2, an e-Delphi technique will be applied. The Delphi survey is a technique that collects the opinions of relevant stakeholders to arrive at a consensus on a topic. Its use is common in the development of COS; the e-Delphi version allows for the process to be carried out online thus enabling greater international participation. The method involves asking stakeholders to complete two or more rounds of the same survey, in which stakeholders rate the importance of each outcome and factor. After each round, the responses are tabulated and redistributed to the participants. The intention is that the iterative process will facilitate consensus on the most important outcomes.

In this project, two rounds of Delphi surveys will be conducted. The DelphiManager software developed by the Core Outcome Measures in Effectiveness Trials Initiative (Liverpool, England - http://www.comet-initiative.org/delphimanager/) will be used to conduct the e-Delphi rounds and analysis.

#### Participants

The panel of participants in the Delphi surveys will consist of three stakeholder groups: (1) women with current or prior GDM; (2) healthcare/public health professionals; and (3) researchers. The recruitment of women with current or prior GDM and professionals will primarily be from Australia, Canada, Denmark, the United Kingdom, and Ireland, whereas recruitment of researchers in the area will have a broader geographical focus, aiming to include participants globally.

Potential stakeholders will be identified through the most appropriate contact methods for each stakeholder group. For women with current or previous GDM, we will use online parenting forums (e.g. www.eumom.ie, www.babycentre.com), clinics, and social media. For healthcare professionals, we will use national and international professional associations (e.g. Irish Nutrition and Dietetic Institute, Diabetes Canada, The Danish Diabetes Association.) Healthcare professionals will be welcome to invite current or former patients with GDM to also participate in the survey. For researchers, we will use international research organizations and networks (e.g. Health Research Board Mother and Baby Network, Diabetes Canada, International Federation of Gynaecology and Obstetrics [FIGO]). Corresponding authors of relevant publications from stage 1 will also be contacted. Participants will be invited to participate in the Delphi surveys via email. Delphi surveys typically involve 20–50 participants, with lower numbers for more homogeneous groups, but we will be taking into account factors such as attrition, contexts, and diversity of responses. We aim to invite at least 150 participants to the Delphi surveys and at least 20 participants in each stakeholder group.

Informed consent will be obtained from participants at the time of registration onto the online questionnaire. Participants will be informed of the study objectives, their right to withdraw, and that data will be handled with confidentiality and anonymized. They may also have any questions answered before registration and consent through a dedicated email address. The importance of participation throughout the whole Delphi process will be emphasized and two email reminders will be sent to non-responding registered participants in each round. The software system will assign each participant a unique identifier allowing for the follow-up and monitoring of attrition throughout the e-Delphi survey rounds.

#### Procedures

On the registration form, participants will be asked to provide information about the relevant stakeholder group or groups applying to them, as well as demographic information such as age group and ethnic background. On the home page of each Delphi round, participants will be provided with plain language summaries on COSs and the Delphi process. Throughout the survey, plain language definitions of each outcome are also provided. Health professionals and researchers will be invited to complete the survey in English. Women with current or prior GDM will have the option of completing the survey in English, French, or Danish.

#### Round 1

In the first Delphi survey round, the full list of outcomes is provided to the participants, who will be asked to provide a score for each outcome. Participants will be asked the question “How important do you think each outcome is to measure in lifestyle interventions to prevent diabetes in women with previous gestational diabetes?” The grading of outcomes is based on the Grading of Recommendations, Assessment, Development, and Evaluations (GRADE) working group [20] and will be on a scale of 1–9; a score of 1–3 represents a lack of importance, a score of 4–6 represents an outcome that is important but not critical, and a score of 7–9 represents a critical outcome. If participants do not feel able to score an outcome, there is an option to select “Unable to Score.” Outcomes will be listed by domain. At the end of round 1 of the survey, participants also have the opportunity to suggest additional outcomes that they deem important, but that are not already listed on the survey.

Participants will be given three weeks to complete the questionnaire. Non-responding participants will receive a reminder email at the beginning of week 2 and week 3.

#### Analysis round 1

The responses from the first round will be collated and summarized using descriptive statistics. The distribution of each outcome score will be computed for the whole Delphi survey and for each individual stakeholder group. Any additional outcomes suggested by participants will be reviewed and discussed by the research team. Outcomes suggested in round 1 that are not part of the original list will be included in the second e-Delphi round together with all the outcomes ranked in the first round.

#### Round 2

Only first round participants will be invited to complete a second survey. They will be presented with the summarized scores for each outcome stratified by stakeholder group and the number of respondents for each score and group. In addition, they will be reminded of their own round 1 score for each item. They will be asked to consider the presented information and invited to rescore each outcome. Changes made to the scoring will be recorded. Any outcomes added after the first round will also be presented and participants asked to score these.

#### Analysis round 2

In the analysis of the second Delphi survey round, the number of participants will be documented and the data will be collated and summarized using descriptive statistics. Distribution of scores will be computed for each outcome and stakeholder group. Determination of consensus for each outcome, by stakeholder group, will be based on definitions used in other COS studies [21–24]. Specifically, it will be defined as ≥ 70% participants scoring 7–9 *and* < 15% scoring the outcome as 1–3. Consensus that the outcome should not be included in the COS will be defined as 70% of participants scoring the outcome as 1–3 and < 15% giving it a score of 7–9. Outcomes for which the distribution of scores differs from this will be classified as “no consensus achieved.”

### Stage 4: Consensus meeting

The face-to-face consensus meeting will be held following completion and analyses of the Delphi survey. The meeting will be held in Halifax, Canada preceding the Diabetes Canada Meeting in October 2018. Participants will include the Co-Principal Investigators (KD, SOR, HTM) respectively from Canada, Ireland, and Denmark as well as internationally recognized GDM clinicians and researchers from Australia (Dr David McIntyre), the United States (Dr Rhonda Bentley-Lewis), and a convenience sample of GDM clinicians, researchers, and women with current or prior GDM representatives from across Canada.

#### Procedures

Before the consensus meeting, participants will be provided with a plain language printed summary of the COS process, the results of the systematic review, a list of all of the original and additional outcomes included in rounds 1 and 2 of the Delphi survey, and the results of the survey (i.e. consensus for inclusion, consensus for exclusion, no consensus; analyses by stakeholder group). This will be followed by a roundtable discussion about the outcomes, the key context and setting attributes identified, including their importance and how to measure these. The roundtable discussion will be conducted in a neutral manner with all outcomes included. Following the discussion, participants will rate every outcome for inclusion (yes or no rating). Any outcome with > 70% “yes” rating will then be discussed before a final rating of outcomes for the COS. The outcomes that receive > 70% “yes” rating in the final ranking will be included in the final outcome set.

## Discussion

With the current lack of a COS for diabetes after pregnancy prevention studies, the development of a COS will enhance opportunities for comparison of future studies and allow for better and larger meta-analyses of the effects. Furthermore, a COS is expected to provide a better understanding of the health behavior change mechanisms used in trials and enhance the quality and assessment of studies by reducing risk of bias and improving reporting. The COS-DAP project will develop a COS for the evaluation of health behavior change interventions seeking to prevent DAP, focusing not only on the affected women’s health, but also the health of their children and spouses. The work will be carried out in a close collaboration with researchers from at least three countries and with the involvement of multiple stakeholders, including healthcare professionals, public health experts, women with prior GDM, and researchers. The inclusion of the perspectives of multiple stakeholders through their involvement in the e-Delphi and consensus meetings increases the chances of the final COS being both applicable and relevant (Additional file 1).

## Trial status

This is an expanded version of the first protocol, which was written to obtain funding from the Canadian Institutes of Health Research. Further protocol modifications will occur if logistical considerations mandate these but will require consensus among the three PIs (SOR, KD, and HTM). Stage 1 (systematic review) and stage 2 (investigators meeting) of the study have been completed and the first round of the e-Delphi began July 2018. The second round of the e-Delphi is expected to be completed by October 2018. The systematic review led to an evaluation of the penetration and participation (PIPE) of completed DAP prevention studies following GDM that report on these PIPE components, which was published early online in April 2018 [25].

## Additional file

Additional file 1: SPIRIT 2013 Checklist. (DOC 123 kb)

## Abbreviations

CICI: Context and Implementation of Complex Interventions
COMET: Core outcomes measures in effective trials
COS: Core outcome set
DAP: Diabetes after pregnancy
DPP: Diabetes Prevention Program
GDM: Gestational diabetes mellitus
GRADE: Grading of recommendations, assessment, development and evaluations
PIPE: Penetration, Implementation, Participation, and Effectiveness
PRISMA: Preferred Reporting Items for Systematic Reviews and Meta-Analyses
RCT: Randomized controlled trials
